# Conflicting priorities: evaluation of an intervention to improve nurse-parent relationships on a Tanzanian paediatric ward

**DOI:** 10.1186/1478-4491-7-50

**Published:** 2009-06-23

**Authors:** Rachel N Manongi, Fortunata R Nasuwa, Rose Mwangi, Hugh Reyburn, Anja Poulsen, Clare IR Chandler

**Affiliations:** 1 Community Health Department, Kilimanjaro Christian Medical Centre, Moshi, Tanzania; 2 Joint Malaria Programme, Kilimanjaro Christian Medical Centre, Moshi, Tanzania; 3 Department of Infectious and Tropical Diseases, London School of Hygiene & Tropical Medicine, London, UK; 4 Department of International Health, University of Copenhagen, Copenhagen, Denmark

## Abstract

**Background:**

Patient, or parent/guardian, satisfaction with health care provision is important to health outcomes. Poor relationships with health workers, particularly with nursing staff, have been reported to reduce satisfaction with care in Africa. Participatory research approaches such as the Health Workers for Change initiative have been successful in improving provider-client relationships in various developing country settings, but have not yet been reported in the complex environment of hospital wards. We evaluated the HWC approach for improving the relationship between nurses and parents on a paediatric ward in a busy regional hospital in Tanzania.

**Methods:**

The intervention consisted of six workshops, attended by 29 of 31 trained nurses and nurse attendants working on the paediatric ward. Parental satisfaction with nursing care was measured with 288 parents before and six weeks after the workshops, by means of an adapted Picker questionnaire. Two focus-group discussions were held with the workshop participants six months after the intervention.

**Results:**

During the workshops, nurses demonstrated awareness of poor relationships between themselves and mothers. To tackle this, they proposed measures including weekly meetings to solve problems, maintain respect and increase cooperation, and representation to administrative forces to request better working conditions such as equipment, salaries and staff numbers. The results of the parent satisfaction questionnaire showed some improvement in responsiveness of nurses to client needs, but overall the mean percentage of parents reporting each of 20 problems was not statistically significantly different after the intervention, compared to before it (38.9% versus 41.2%). Post-workshop focus-group discussions with nursing staff suggested that nurses felt more empathic towards mothers and perceived an improvement in the relationship, but that this was hindered by persisting problems in their working environment, including poor relationships with other staff and a lack of response from hospital administration to their needs.

**Conclusion:**

The intended outcome of the intervention was not met. The priorities of the intervention – to improve nurse-parent relationships – did not match the priorities of the nursing staff. Development of awareness and empathy was not enough to provide care that was satisfactory to clients in the context of working conditions that were unsatisfactory to nurses.

## Background

Patient, parent or guardian satisfaction with health care is now seen as central to the performance of health services [1]. Satisfaction with health care delivery affects likelihood of compliance with treatment [2], likelihood of absconding during admission and willingness to pay for treatment [3], as well as overall usage [4] and demand for services [5,6].

Interpersonal aspects of care have been identified as key to community satisfaction with health services in many settings [7-9], and may even outweigh the importance of perceived technical competence. For example, among community members in Tanzania, "receiving the 'right' drugs from a rude health worker represented a poorer quality of care than receiving the same drugs from a polite worker, and perhaps was even poorer than receiving the 'wrong' drugs from a polite worker" [10].

Improving usage of health facilities by communities and quality of care delivered by health workers is essential if targets for better health are to be achieved in developing countries [11]. Improving interactions between health workers and clients, particularly nursing staff, who in many settings are the face of health care delivery, is key to increasing satisfaction with health care [12].

Participatory research, a popular approach to behaviour change, is a process whereby insiders collaborate with outside researchers as equal partners to explore current action with the intention of generating change [13]. A series of workshops has been designed based on this approach with the aim of improving health worker-client relationships in developing countries.

The workshop series is called Health Workers for Change (HWC) and encourages health workers to critically examine the way they relate to clients, with a particular gender emphasis, and the factors that influence this relationship. The workshops are designed to empower and transform participants, motivating them to take constructive action [14].

The workshops are described in a manual published by the World Health Organization [15]. The series has undergone evaluation for acceptability in four different African settings [16] and evaluation for effectiveness in seven different primary health care settings. It was found to result in positive changes in terms of reduced time spent at facilities by clients at five sites; improved interactions between health workers and clients at four sites; and improved interactions between staff at four sites where problems were discussed more openly and staff took the initiative to solve problems themselves [17]. The effectiveness of the workshop series as an intervention for nurses working in a hospital inpatient setting has not yet been explored.

### Study objective

We aimed to assess the ability of the HWC workshop series to improve the quality of the relationship between nurses and parents on the paediatric ward of a regional hospital in Tanzania where low quality of technical and interpersonal care had previously been reported as part of an assessment of paediatric care at 13 hospitals in northeast Tanzania [18,19]. In addition to the impact of the intervention, our study evaluated the process of the intervention and ventured to understand factors affecting the intended outcomes of the intervention.

## Methods

### Study design

The study evaluated the effect of a workshop intervention, Health Workers for Change, on nurse-parent relationships on a paediatric ward in a busy regional hospital in Tanzania. The evaluation used before-and-after questionnaires with parents/guardians and two after-intervention focus groups with nursing aides and trained nurses to assess the effect of the intervention.

### Study setting

The intervention was implemented and evaluated in a regional hospital serving more than 110 000 outpatients and 20 000 inpatients each year (data from 2007 hospital records). The hospital has 13 wards, including two linked wards for paediatrics, wards 4A and 4B, with a daily average of 50 and 17 paediatric inpatients staying at each, respectively.

The hospital employs 427 staff members, of whom 142 (33%) are trained nurses and 188 (44%) are nursing attendants. In all, 31 nursing staff worked on the paediatric wards at the time of the study; 13 were trained nurses (they had completed four years' training as nurse midwives or nursing officers) and 18 were nurse attendants (they had completed one year of training pre-service).

The median time in post was 21 years (range 10 months-37 years) and median duration on the paediatric ward was seven years (range nine months-22 years). The average salary of nursing attendants was USD 80, with trained nurses earning an average of USD 290. The area has low and seasonal malaria endemicity. The study took place during the peak malaria season from May to July 2007.

### Study population

The study population consisted of all nurses on the paediatric ward. Twenty-nine of these nurses participated in the workshops and 24 in the post-workshop FGDs, six months later. The nurses were assessed for interpersonal care by two populations of parents: 144 parents with children on the ward prior to the workshops and a further 144 with children on the ward six weeks after the workshops. As most children on paediatric wards are accompanied by mothers, we refer to all parents/guardians as mothers in this paper.

### Workshops

The workshops followed the format set out in the Health Workers for Change manual[15]. This involved six workshops that took place over a period of three weeks, with two, two-hour sessions per week. The workshops addressed the following topics: (1) "Why I am a health worker", (2) "How do our clients see us?", (3) "women's status in society", (4) "unmet needs", (5) "overcoming obstacles at work", and (6) "solutions".

The hospital and regional administrations were consulted and gave support to the project. Nurses of all levels, including nursing attendants, who were working on the paediatric ward were invited by letter to attend an initial meeting to introduce the workshops and to arrange convenient dates and times for the workshops. The workshops took place in a self-contained building at the hospital site. Participants were provided with refreshments and a return fare from their home on the days of the workshops. Two researchers ran each of the workshops (RM & FN), which included various exercises such as role plays, paper-and-pen small-group exercises, narratives and discussions. The workshops were conducted in Kiswahili, with notes on the proceedings and verbatim quotes recorded by hand. All participants gave informed consent to participate and were aware that the process was being evaluated.

### Data collection methods

#### Parent satisfaction questionnaire survey

##### Questionnaire design

We designed a parent satisfaction questionnaire for use before and after the workshops to evaluate their effect. There is no standardized patient satisfaction measurement tool for developing countries.

Picker survey instruments for assessing patient experiences of health care are gold standard surveys widely used across the developed world [20]. The Picker adult inpatient questionnaire, comprising 40 items, has been reduced to a core set of items that have been validated for use across different settings, termed the Picker Patient Experience 15, or PPE-15 [21]. We adapted the reduced questionnaire for nursing care for inpatient paediatrics with additional context-specific questions developed from the focus-group discussions with mothers.

Following Jenkinson et al. [21], we conceptualized the questionnaire in terms of each item as a problem (Table 1). Each was then phrased as a question, with a range of possible responses, for example:

**Table 1:** Problems identified by the questionnaire

**Item**	**Item content**
1.	Not shown where to wash, cook and use the toilet

2.	Ward and toilets not cleaned often enough by staff

3.	Mothers expected to clean the ward and toilets themselves

4.	Not given enough information about cause of illness

5.	Nurses' answers to questions not clear*

6.	Staff gave conflicting information*

7.	Nurse didn't discuss anxieties or fears*

8.	Nurses sometimes talked as if I wasn't there*

9.	Not sufficiently involved in decisions about treatment and care*

10.	Not always treated with respect and dignity*

11.	Not easy to find someone to talk to about concerns*

12.	Not clear whom to ask for medical assistance on the ward

13.	Nursing staff unavailable when needed on the ward

14.	Nursing staff rude/unhelpful when asked for medical assistance on the ward

15.	Test results not clearly explained

16.	Nurses performed medical tasks poorly

17.	Child not told about what was happening when undergoing procedures

18.	Staff did not do enough to control pain*

19.	Purpose of medicines not explained*

20.	Not told about medication side effects*

*Indicates taken from PPE-15

Did nurses give you enough information about your child's illness in a way that you could understand?

[1] Yes, definitely

[2] Yes, to some extent

[3] No

The answers for each question could then be given a binary value of the presence or absence of a problem. The resulting survey was translated and back-translated into Kiswahili by a team of translators. A copy of the full questionnaire is available from the authors.

Content validity of the questionnaire was strengthened by the initial FGD results and construct validity was measured using the discriminance, or the "extreme groups" method (the extent to which the questionnaire produces results that concur with the underlying theoretical construct) [22]. Reliability was measured with Cronbach's coefficient, showing the average correlation among items in the questionnaire.

##### Questionnaire sample

We used data from a 2005 patient satisfaction study at this hospital, when 36% of 42 mothers interviewed stated that they found the nursing staff to be polite or helpful [18], to estimate the sample size needed to detect an increase in satisfaction of 50% with 80% power and 95% confidence. The result was 143 mothers each before and after the workshops, allowing for 10% unusable data.

The primary parent or guardian of every child under five years of age admitted to the paediatric ward within 24 hours of the time of the survey was eligible for inclusion in the survey sample.

##### Interview procedure

With the permission of the staff on the ward, parents of eligible children were approached and, after oral informed consent was obtained, were interviewed by an experienced researcher (FN) in a secluded area of the ward.

##### Analysis

Data were double-entered into Microsoft Access 2007 and analysed by means of STATA 10 (Statacorp, Texas, United States of America). Analysis of the questionnaire used a dichotomous problem score, indicating either the presence or absence of a problem, with a simple additive scoring algorithm, following Jenkinson et al. [21]. Z-tests were used to compare data before and after the workshops, including demographic variables, individual problems identified by respondents and additive problem scores.

#### Focus group discussion

##### Conduct of FGDs

Two FGDs with (1) nurse attendants and (2) trained nurses on the paediatric ward were conducted six months after the first post-workshop visit. The FGDs were conducted by one facilitator (RM, medical doctor and social scientist), one assistant (FN, social science research assistant) and one experienced note taker.

After giving introductory information and obtaining consent from participants, the moderator followed a question guide to explore current relationships between nurses and mothers, the roles and expected roles of each, barriers to good relations and any changes since the workshop series. The discussions continued on each topic until no new information was gained.

Discussions were held in Kiswahili and were tape-recorded, with notes taken of verbal and non-verbal responses and as to which participants were speaking. These notes were expanded immediately after each FGD.

### Data management and analysis

Records from workshops and the FGDs were transcribed and translated and then checked by FN. The transcripts and discussion notes were read line by line and coded by CIRC and RNM according to ideas represented in each section of text. These 'idea codes' were then grouped together as themes using NVivo version 7.0 (QSR International, Cambridge, Massachusetts, United States of America). Themes were discussed within the research team to explore meanings and arrive at a consensus of interpretation of the data.

## Results

We present the proceedings of the workshops, followed by the results of the before-and-after parent questionnaire evaluation and the post-intervention nurse focus-group discussion.

### Workshops

Between 26 and 29 of the 31 nurses scheduled to work on the paediatric ward attended each of the workshops, with roughly equal numbers of trained nurses and nurse attendants. A summary of issues raised at each of the first five workshops is shown in Table 2.

**Table 2:** Workshop summaries

**Workshop**	**Summary of responses**
1. Why I am a health worker	"It was to help my family and the community as a whole" "To give service and comfort to the sick" "I was attracted by the white uniforms, stethoscope and pushing a trolley with medicine" "I wanted to improve health services as it was bad in the areas around us" "It was a good job that was reliable and had more value than others" "It was the only way to get employed" "I wanted to know about different diseases and prevention"

2. How do our clients see us?	There is poor cooperation between doctor and nurse. Doctors are our bosses. They do not respect nurse attendants. They want to be attended by nurses in white or by doctors. They feel we don't care because of staff shortages.

3. Women's status in our society	Women have fewer educational opportunities. Women often do not know their rights. Women may work harder than men. Men are respected more than women. Women and men may be ignored if they are poor.

4. Unmet women's needs	Women need to be empowered through health education. Women need education about their rights in making decisions. Tolerance must be promoted between men and women.

5. Overcoming obstacles at work	Low salary Inadequate equipments. No respect between staffs and between patients. Shortage of staffs. Fear of infections.

During the workshops, nurses acknowledged that they sometimes had a poor relationship with parents of children on the ward. For example, in the role plays nurses demonstrated that when mothers asked them questions on the ward they might become rude and unhelpful. A nurse role-playing a mother asking when her child's intravenous line would be attended to elicited as a typical response from other nurses "It is not your job to remind me" and "Wait for the nurses in the next shift".

### Motivation and respect: ideals and reality

In workshop discussions, many nurses described altruism and achieving community respect as reasons for choosing their profession. However, many were disappointed with the reality of their work, often entailing poor relationships with patients and their mothers.

"Our working environment is very different from what we expected and this situation contributes to the use of harsh language, hating our job and not working hard" (trained nurse, TN, workshop one, W1).

Several staff members said that their personal ideals had been replaced by a degree of cynicism towards their work. Many felt this was an inevitable (and to an extent justified) result of the low levels of recognition or reward that they received from their work. This was exemplified in a role play depicting a rich woman arriving at the ward, which led to the following comments by participants:

"I was happy seeing my friend. I was aware that there was a patient to attend but my salary is too small. I couldn't even have tea in the morning, so my friend was my hope when I saw her. The poor sick woman had nothing to help me. I had to leave her waiting" (nurse attendant, NA, W1).

"A rich person is always served first in the hospital because we expect to get something from her/him. And this is all because the low income makes us easily tempted with small things, like soda" (TN, W1).

"The administration does not care for the workers so it turns someone to be irresponsible" (TN, W1).

Nurses described feeling undermined by a lack of respect from colleagues, particularly from senior colleagues who were reported to speak harshly to them in front of patients.

"Patients ignore me, I get angry, and I give poor service and use bad language, because the doctor has already shown I am not competent" (TN, W3).

Nurse attendants also felt undervalued by patients, a feeling that was enhanced by their orange uniform (perceived as "non-medical") compared to the white uniform of nurses.

"If we help nurses to attend patients, some patients refuse and say 'I want to be attended by a nurse in a white uniform' so there is no trust in us" (NA, W1).

"The difference in uniforms results in disrespect between us and patients. When I want to attend him/her they openly say I want a nurse with a white uniform. This makes me feel inferior and so instead I will be using harsh words and not giving a quality service" (NA, W5).

The superiority of higher cadres of health worker over lower levels was brought out in a role play designed by participants in a workshop to illustrate how this affected the standard of care.

Scene 1: A very sick woman enters the ward assisted by a care-taker. She is glanced at by the doctor, who calls for the nurse in charge to show the patient a bed. The nurse in turn glances at the patient and calls for the nurse attendant, currently cleaning the ward, to show the patient to a bed.

Scene 2: The doctor listens to the patient's history from the care-taker and calls for the nurse to administer a drip. The nurse calls for the attendant to administer the drip.

Nurses reflected that it was often difficult to approach senior staff for clinical advice on patients, as these were likely respond in a dismissive way towards junior staff.

### Salary and working conditions

Low wages and lack of allowances were frequently cited as reasons for low motivation towards work and for giving poor service.

"The salary is low. I am not satisfied when I get to work. I only think of how to get money. I ask patients to give me some money so that I give them quality service or I bring things to sell around what I get will help boost my life. So instead of helping the sick I just think of a business to give me income" (TN, W5).

"If there was allowance for working long hours I would have been the most hardworking of staff" (TN, W5).

In addition to financial and status issues, nurses identified other restrictions in their working environments that led to poor relationships with mothers (Table 3). These included a lack of equipment: "There is not enough equipment to make our work good... And unavailability of gloves makes attending the patient poor because you can't touch him/her for the fear of disease transmission" (NA, W5), and a perception of unfair decisions made by managers: "Some people are promoted and some never get that chance" (TN, W5).

**Table 3:** Problems leading to poor attitudes towards patients and carers

**Problem identified by nurses during workshop 5**	Average rank of each problem*
Respect from colleagues and carers	4.75
Low salary	2.86
Inadequate equipment	1.36
Shortage of staff	1.25
Infection risk	1.25
Working overtime	1.11
No allowances	1.00
Long working hours	0.93
No promotions	0.21
No in-service training	0.07

*All nurses ranked their top five most important problems from 5 (most important) to 1 (least important). The ranks for each problem were summed and divided between the 28 participating nurses.

### Solutions

Participants reviewed the discussions from the previous five workshops in a final session designed to stimulate solutions. These are presented in Table 4 and are divided between action points to improve relations with mothers through internal nursing dynamics, and points to improve conditions of work affected by external forces, i.e. hospital administration.

**Table 4:** Solutions agreed at workshop six

	**Solution action points**	**Implemented by follow-up visit at 6 weeks**
*Improvements internal to nursing group*		

Maintain cooperation	• Arrange a meeting with doctors to explain the importance of working together and respect for each other. • Have regular meetings together to maintain respect and address issues.	• Meetings had taken place weekly with nurses and doctors, identifying respect as an issue particularly with nursing attendants. Used as a forum for problems with work.

Prevent infections	• Staff training on disease prevention. • Disseminate education on disease prevention to patients and mothers.	• 3/4 nursing staff had attended disease prevention training run by the MoH at the hospital.

Respect each other	• Be close to fellow staff. • Help each other on job allocation. • Observe punctuality.	• Improved assistance between staff was reported, although shortages persisted.

Work conscientiously	• Be active at work without thinking of low salaries.	

*Improvements via external forces*		

Low salary	Request from employer at staff meeting with Regional Administrative Secretary.	• Incremental increase agreed to be paid.
		
Allowances		• Not agreed. No budget.
		
Shortage of staff		• Problem persists.
		
Risk allowance		• Not agreed as not in MoH plan.
		
Inadequate equipment		• Request taken forward, but no action at this point.
		
Transport for staff		• Not agreed. Not allocated in government budget.
		
Staff house		• Not affordable for all staff, attributed to government budget.

A follow-up visit was made six weeks after the last workshop to find out what action had been taken by the nurses in the study. Researchers were not part of the process of requesting changes at the hospital level, as this might have biased any response from the administration.

At the follow-up visit, nurses reported that the action points had mostly been addressed, although largely without the desired outcome. Meetings were reported to have been held among the ward staff to address issues of respect and assistance to colleagues, and some improvement was reported. However, the result of the meeting with the Regional Administrative Secretary was less successful. Many of the issues raised were reported to be in the control of the government rather than the region and therefore could not be addressed locally.

### Evaluation I: Parent satisfaction

In all, 288 parents/guardians participated in the satisfaction survey, 144 over a one-week period before the workshops and another 144 six weeks after the end of the workshops. Demographics of the questionnaire respondents were not significantly different in the two surveys: in 95% cases the respondent was the mother; the median age of the respondent was 26 years (IQR 23, 30). The demographics and diagnoses of children were almost identical at the two survey times. The median age of children was 12 months (IQR 7, 24); the median length of stay at the time of interview was three days (IQR 1,4); 38% children were diagnosed with malaria, 33% with diarrhoea and 22% with pneumonia.

Cronbach's coefficient was high, at 0.85 for the baseline questionnaire and 0.77 for the post-intervention questionnaire, suggesting that variation in scores is more likely to be due to variation in true differences rather than measurement error. Validity testing by means of the discriminance method suggested that the questionnaire was valid; for example, mothers who reported having to clean the ward or toilets themselves were statistically significantly more likely to cite problems (p < 0.001 for both surveys).

**Figure 1:**
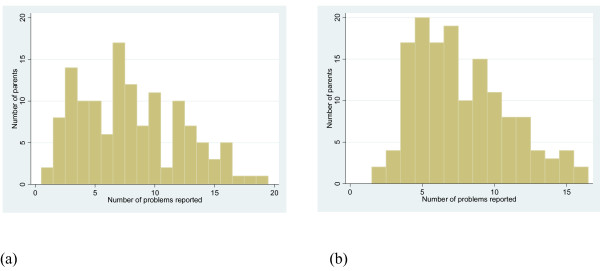
**Number of problems reported at (a) the baseline survey and (b) the post-intervention survey**.

Analysis of parent satisfaction questionnaires showed frequent problems reported both before and after the intervention (Table 5). Overall, the mean percentage of mothers reporting each of 20 problems was not statistically significantly different after the intervention, compared to before it (38.9% versus 41.2%). However, the number of problems reported by individual mothers did decrease overall, with a shift in this distribution to the left (Figure 1).

**Table 5:** Dichotomous problem score results before and after the intervention

**Item**	**Item content**	**% reported as a problem: Baseline**	**N**	**% reported as a problem: Post Workshop**	**n**	**z-test p-value**
1.	Not shown where to wash, cook and use the toilet.	67.8	143	55.6	144	0.032

2.	Ward and toilets not cleaned often enough by staff.	27.8	144	22.9	144	0.343

3.	Mothers expected to clean the ward and toilets themselves.	31.7	142	35.4	144	0.515

4.	Not given enough information about cause of illness.	51.4	144	63.9	144	0.032

5.	Nurses' answers to questions not clear.	29.2	144	30.6	144	0.797

6.	Staff gave conflicting information.	18.1	144	3.5	144	< 0.001

7.	Nurse didn't discuss anxieties or fears.	45.1	144	9.7	144	< 0.001

8.	Nurses sometimes talked as if I wasn't there.	31.0	142	61.8	144	< 0.001

9.	Not sufficiently involved in decisions about treatment and care.	96.5	144	99.3	144	0.099

10.	Not always treated with respect and dignity.	21.0	143	19.4	144	0.746

11.	Not easy to find someone to talk to about concerns.	42.4	144	13.9	144	< 0.001

12.	Not clear who to ask for medical assistance on the ward.	21.5	144	13.2	144	0.062

13.	Nursing staff unavailable when needed on the ward.	27.8	144	31.3	144	0.518

14.	Nursing staff rude/unhelpful when asked for medical assistance on ward.	36.8	144	54.2	144	< 0.001

15.	Test results not clearly explained.	45.8	144	38.2	144	0.189

16.	Nurses performed medical tasks poorly.	30.6	144	16.7	144	0.006

17.	Child not told about what was happening when undergoing procedures.	35.4	144	22.2	144	0.013

18.	Staff did not do enough to control pain.	44.1	102	41.7	127	0.717

19.	Purpose of medicines not explained.	47.1	138	56.3	144	0.124

20.	Not told about medication side effects.	75.7	144	88.9	144	0.003

21.	Mean of all questions	41.2	144	38.9	144	0.690

Analysis of specific components of the satisfaction questionnaire found some improvements, although some stayed the same and some aspects appeared to worsen (Table 5, Table 6). The items with the most statistically significant improvement were those that measured the responsiveness of the nurses, for example in discussing anxieties (problem for 45% mothers fell to 10%), being able to find someone to talk to about concerns (problem for 42% fell to 14%), telling the child about his or her procedures (problem for 35% fell to 22%), and, more technically, being more careful when performing medical tasks such as injections and taking blood (problem for 31% fell to 17%).

**Table 6:** Questions showing better, worse and the same responses

**Problem level reduced (i.e. improvement)**	**Problem level increased (i.e. worse)**	**Problem level stayed same**
Not shown where to wash, cook, toilet	Not given enough info about cause of illness	Ward and toilets not cleaned by staff

Staff gave conflicting information	Nurses sometimes talked as if I wasn't there	Mothers expected to clean the ward and toilets themselves

Nurse didn't discuss anxieties or fears	Nursing staff unhelpful when asked for medical assistance on the ward	Nurses' answers to questions not clear

Not easy to find someone to talk to about concerns	Not told about medication side effects	Not sufficiently involved in decisions about treatment and care

Not clear who to ask for medical assistance on the ward		Not always treated with respect and dignity

Nurses performed medical tasks poorly		Nursing staff unavailable when needed on the ward

Child not told what was happening when undergoing procedures		Test results not clearly explained

		Staff did not do enough to control pain

		Purpose of medicines not explained

Improvements were also made in aspects of role definition: mothers were more likely to have been shown where to wash, cook and use the toilet (problem for 68% fell to 56%) and had fewer problems with knowing whom to ask for assistance on the ward (22% fell to 13%). Fewer mothers reported problems with receiving conflicting information (18% fell to 4%) but more mothers reported that they had not been given enough information about the cause of illness (51% rose to 64%).

In addition, attitudes of nurses towards mothers did not appear to have improved: mothers reported nurses talking as if they weren't there (31% rose to 62%) and being rude or unhelpful when asked for medical assistance on the ward more often (37% rose to 53%); the proportion reporting being treated with respect and dignity did not increase (21% before and 19% after).

### Evaluation II: Follow-up FGD with nurses and attendants

During the workshops we noticed differences in opinions and some tension between nursing attendants and trained nurses. We therefore conducted evaluative FGDs with each of these groups separately, six months after the last follow-up visit. All nurses still working on the paediatric ward (30) were invited, and 24 accepted, 15 for the nurse attendants' FGD and nine for the trained nurses' FGD. The nurse attendants were younger, between 28 and 57 years, while trained nurses were aged between 41 and 58 years.

#### Attitude change

The response to the workshops was overwhelmingly positive among both nursing attendants (NA) and trained nurses (TN), although barriers to good relationships between staff and between staff and patients remained. Participants reported taking on a more positive attitude towards work, and empathizing more with mothers.

NA08 "After the workshop there is no laziness. For example when a child arrives seriously sick, I take the tests to the laboratory and ask them to work on it fast so that I can go and give the child medication. The seminar has helped us to change and to work hard".

TN01 "For us as nurses, the workshops we did helped us as we now have a close relationship with our clients, we have increased love to them, we have time to listen to their complaints. The workshop helped us to correct ourselves [*kujirekebisha*]".

#### Relationships between nursing staff

Nurse attendant participants frequently attributed their improved attitudes to improvements in their relationship with the nurses since the workshops.

NA09 "After the workshops we planned how to cooperate between us and nurses on high grades. We sit and discuss if there are problems and how to overcome them. Therefore generally we are all in the same truck".

NA03 "We now work in cooperation and help each other. So, when we get in the ward they do the dusting and we mop. Thereafter we all do something else together. Therefore the workshop has helped us much with working in cooperation".

NA12 "I can ask the nurse officer to help me do something and they do it with all their heart".

Improved cooperation between ward staff was also perceived by the trained nurses, although some indicated that this was a temporary solution to staff shortages and it resulted in an erosion of their status.

Facilitator: "How is your relationship with nurse attendants?"

TN08: "Our relationship has become so good there is no distinction between work for attendants and for nurses. We all work together, and they do not complain anymore..."

TN02: "In addition to that there is a shortage of staff... those who are to sweep are also expected to help in injecting the patients; this is very much discouraging. There should be an increase of staff. But generally the relationship is good between us and we do our best to support them with the aim of giving good service to patients."

Nurse attendants also felt that greater cooperation between ward staff resulted in improved care and friendlier relations with mothers:

NA15: "Good cooperation helps us work better with the mothers and between us staff. Mothers see that we care about them because when they come to ask or need help we are always there for them. And with us attendants if we see that there are cases we can't attend, we are free to ask the more qualified nurses and they help. Before they were not helpful; if you asked for assistance they left everything to you... I am truly happy with how the mothers act to us. Before if we asked them for a favour they would leave the ward and sit outside, but now they are ready even to help straighten the beds".

#### Barriers remaining

In spite of these improvements, participants were aware that further improvements were possible. For nurse attendants, problems in their relationships with trained nurses remained, and for trained nurses, problems with doctors remained.

NA08: "We [nurse attendants] come for the night shift alone with no trained nurses. Only two nurse attendants and you find the ward has 50 patients..."

Facilitator: "So they are not assigned for the night shift?"

NA03: "They come, but they stay in the office. If patients come with complications, that's when we go to call them."

NA01: "And if you ask them for help they tell us: 'If you couldn't do it, what do you think I can do?' ... If you ask her to help to get a vein she'll say: 'If you couldn't get one, how can I? What do you want me to do?"'

TN02 "I always argue with the doctors at the OPD [outpatient department], because if you take a child there they say 'I don't understand' or 'I'm busy', as if that is my child. And I believe that would happen even if I brought my own child. Therefore services become bad from the doctors. They have a lot to do...If we call a doctor for one serious case, he is not ready to help the others who just need discharging. He will keep saying 'I'm called for only one person'. This is discouraging".

Many of the difficulties in the working environment discussed in the workshops remained at the time of the FGDs, cited as preventing nurses from maintaining good relations with mothers. These included low salaries, long shifts and lack of consideration from the administration in terms of allowances or refreshments for night duties.

Facilitator: "What obstacles do you see in maintaining the relationships with the mothers?"

NA08: "Our income can be an obstacle in maintaining our good relationship with mothers, because we get very low salary, there is a lot of work to do, we work overnight and it is so tiring. There is no tea, no bite to eat. I leave my home at 4 p.m. and I come from [several miles from the hospital]. After the shift I leave at 8 a.m. and get home when it's 1 p.m. When I return in the evening I'm still tired, so if a mother asks me something I automatically respond badly."

TN01: "Because the salary is low, when I come to work I think that I have no school fees for children or that I didn't get even a cup of tea at home. My efficiency at work becomes low and therefore there is not good service to patients."

TN08: "If one is given their right [financial compensation] then they automatically work hard. With me I eat my last meal at 1 p.m. and I come for work at 6 p.m. I stay starving all through the night, so I don't do my job well. I suggest we should be given at least an allowance so I can buy something to eat."

#### Perception of power

While the issue of low salaries and lack of allowances had been raised with the Regional Administrative Secretary by the nursing staff after the workshops, participants reported no progress, and both nursing assistants and trained nurses feared raising the topic again. As a result they asked the research team to present their views to managers.

Facilitator: "Are there any other questions? I am finished from my side."

NA01: "There is a question and that is: How are you going to help us with this issue of low salary?"

NA05: "Doctor, I have an addition to that: those who are working in the office are paid for extra hours, but we are not. This is all because we don't have representatives. So I think through you and the team you can help, because in the office they are paid and they are attendants like us. If they extend for 30 minutes, then they sign in the book and they are paid. Please help! That doesn't happen to us because we have no representatives."

TN04 "We are afraid to request for things. Because we are the lower people, nothing will work. I want to ask where all the funds are going"

Trained nurses felt that failure of the government to fund carefully, especially for consumables and drugs, often resulted in conflict between mothers (who expected a free service but were then asked to go out and buy supplies) and nurses (who were left in the position of being suspected of corruption).

TN08: "They said children are treated for free, so mothers think we are asking for bribes for services. It should be advertised as it is. When a child is brought by her mother and the mother is told go and buy syringes and medication, they feel oppressed. If it is said services are free, let it be so; equipment should be available".

TN08: "We shouldn't have to see people are moving to Botswana. They [the government] should improve our working condition so that we stay in our country..."

TN02: "Our Government should look for a way of improving its services. You may find that there are no syringes, no drugs: What quality service do you expect us to give? They should improve things."

TN08: "We need to be given night allowance, because at night we do the overall supervision, answerable to cases that might occur. The government should think of us getting night allowance. It is our right – we don't work as Samaritans ... we want payments. This should be improved".

## Discussion

Tanzania's Health Sector Strategic Plan of 2003–2008 has focused reforms towards the delivery of good-quality health services and meeting clients' satisfaction [23]. This study found that a participatory research approach to improve relationships between nursing staff and parents or guardians of patients on the paediatric ward of a busy regional hospital in Tanzania had limited success.

Overall, improvements were made in the responsiveness of nursing staff to the needs of mothers, but the majority of the factors identified during the workshops as hindering positive relationships remained after the workshop series had finished. The participants in this study may have had less power to affect their working environment in comparison with other, less complex, health facility settings where the Health Workers for Change approach has been effective [17].

Our results suggest that participants were successful in critically analysing their own actions and the workshops may have promoted greater empathy towards mothers. However, this did not translate into better interpersonal relationships as perceived by mothers. The mismatch between parent and health worker priorities in this setting has been reported elsewhere [19].

Several significant barriers were perceived by nurses to interfere with their ability to initiate change. Franco et al. [24] defined motivation in the work context as "an individual's degree of willingness to exert and maintain an effort towards organisational goals" and conceptualized motivation among health workers in low- and middle-income countries in terms of "will do" and "can do" within the context of their personal characteristics, working environment and the broader societal context.

Health workers may be able to perform but unwilling, due to lack of personal impetus or environmental incentives. Similarly, they may be willing but unable to perform well, due to lack of knowledge or environmental constraints. For a long time health workers, given their assumed willingness or altruism in helping patients, were categorized in terms of "can't do", with interventions to improve health care focusing on training to improve knowledge and skills, together with somewhat sporadic environmental improvements in terms of equipment. Few improvements have been made following this approach [25,26].

Health workers were re-conceptualized during the late 20^th ^century as potentially self-interested, removing the "will do" assumption in health policy and leading to questions about how to motivate individual health workers [27]. Preferential treatment for certain patient groups exemplifies this, and our finding of differential treatment for richer clients echoes findings about nurses in another hospital setting in Ghana [28]. In addition, "can do" began to be seen as more complex than knowledge and equipment: the importance of relationships between staff and between staff and communities [29], and organizations' role in managing both human resources and physical resources is increasingly recognized [11,30], together with broader effects of health systems and medical culture on health worker "can do" and "will do" [31].

Our findings suggest that while willingness and attitudes – the "will do" element – may be amenable to change, support is needed from the wider organization for this to be achieved. Nursing staff perceived major problems with their work environment that were prioritized over the need for good relationships with clients, including low salaries with inadequate allowances for extra work, and a lack of respect and support from administrative and more senior health staff.

Salary and relationships with administrators and supervisors or peers are classified by Herzberg [32] as "hygiene factors", necessary to be fulfilled in order to improve performance through "motivating factors" such as achievement, responsibility and personal growth. This theory has been supported by findings from developing-country settings [29,33] and suggests that an approach that improves the "will do" component of motivation may be more effective where hygiene factors are fulfilled.

We surmise that increased responsiveness from the hospital administration, and the health sector more broadly, to the needs of existing nursing staff is needed before changes in their interactions with clients can be expected. Empathy is not enough.

### Limitations

Participatory research is strongly determined by the attitudes of the researchers [34], and this may have affected the impact of the workshops. The results are also specific to the context of the study hospital; further research in other hospital settings and run by other researchers may support or conflict with our findings. Any changes in attitudes or behaviour may not be attributable to the workshop, and although the nursing staff was asked to differentiate changes due to this study and other activities in the hospital, responses of mothers could reflect other factors. No other interventions with similar aims were ongoing at the time of the study, but parental responses could be subject to changes in staff rotas at the times of the questionnaires. The questionnaire instrument was not validated, although attempts were made to improve validity and reliability. The problem-based approach to the questionnaire may avoid classic problems of positive ratings in satisfaction surveys and insensitivity to problems with the specific processes that affect the quality of care delivery [35,36].

## Conclusion

The aim of the intervention was to improve nurse-parent relationships, previously identified as contributing to poor delivery of technical and interpersonal care. We found that despite the use of an evidence-based participatory approach to tackling this problem, we had little success in achieving this goal. Our evaluation suggests this may be because the priorities of the nursing staff did not match those of the intervention. We conclude that until nurses' needs in their working environment are met, it is unlikely they will be able to shift focus to the needs of parents.

## Competing interests

The authors declare that they have no competing interests.

## Authors' contributions

All authors contributed to the conception and design of the study; RNM and FN carried out the workshops, FGDs and survey; RNM and CIRC carried out qualitative analysis of transcripts; CIRC conducted statistical analysis; all authors contributed to the interpretation of the data and to drafting the manuscript. All authors read and approved the final manuscript.

## References

[B1] World Health Organisation (2000). World Health Report 2000. Health Systems: Improving Performance. Geneva.

[B2] Deyo RA, Inui TS (1980). Dropouts and broken appointments. A literature review and agenda for future research. Med Care.

[B3] Hanson K, McPake B, Nakamba P, Archard L (2005). Preferences for hospital quality in Zambia: results from a discrete choice experiment. Health Econ.

[B4] Ndyomugyenyi R, Neema S, Magnussen P (1998). The use of formal and informal services for antenatal care and malaria treatment in rural Uganda. Health Policy Plan.

[B5] McPake B (1993). User charges for health services in developing countries: a review of the economic literature. Soc Sci Med.

[B6] Wouters AV (1991). Essential national health research in developing countries: health care financing and the quality of care. Int J Health Planning and Management.

[B7] D'Ambruoso L, Abbey M, Hussein J (2005). Please understand when I cry out in pain: women's accounts of maternity services during labour and delivery in Ghana. BMC Public Health.

[B8] Lule GS, Tugumisirize J, Ndekha M (2000). Quality of care and its effects on utilisation of maternity services at health centre level. East Afr Med J.

[B9] Uzochukwu BS, Onwujekwe OE, Akpala CO (2004). Community satisfaction with the quality of maternal and child health services in southeast Nigeria. East Afr Med J.

[B10] Gilson L, Alilio M, Heggenhougen K (1994). Community satisfaction with primary health care services: an evaluation undertaken in the Morogoro region of Tanzania. Soc Sci Med.

[B11] World Health Organisation (2006). The World Health Report 2006: Working Together for Health. Geneva.

[B12] Alsop-Shields L (2002). The Parent-Staff Interaction Model of Pediatric Care. J Pediatr Nurs.

[B13] Yeich S, Levine R (1992). Participatory Research's Contribution to a Conceptualisation of Empowerment. J App Soc Psych.

[B14] Haaland A, Vlassoff C (2001). Introducing Health Workers for Change: from transformation theory to health systems in developing countries. Health Policy Plan.

[B15] Fonn S, Xaba M (1995). Health Workers for Change, a Manual to Improve Quality of Care. Women's Health Project and TDR/WHO (TDR/GEN/1952).

[B16] Fonn S, Mtonga AS, Nkoloma HC, Bantebya Kyomuhendo G, daSilva L, Kazilimani E, Davis S, Dia R (2001). Health providers' opinions on provider-client relations: results of a multi-country study to test Health Workers for Change. Health Policy Plan.

[B17] Onyango-Ouma W, Laisser R, Mbilima M, Araoye M, Pittman P, Agyepong I, Zakari M, Fonn S, Tanner M, Vlassoff C (2001). An evaluation of Health Workers for Change in seven settings: a useful management and health system development tool. Health Policy Plan.

[B18] Reyburn H, Mwakasungula E, Chonya S, Mtei F, Bygbjerg I, Poulsen A, Olomi R (2008). Clinical assessment and treatment in paediatric wards in the north-east of the United Republic of Tanzania. Bull World Health Organ.

[B19] Mwangi R, Chandler C, Nasuwa F, Mbakilwa H, Poulsen A, Bygbjerg IC, Reyburn H (2008). Perceptions of mothers and hospital staff of paediatric care in 13 public hospitals in northern Tanzania. Trans R Soc Trop Med Hyg.

[B20] Picker Institute (1999). Implentation manual.

[B21] Jenkinson C, Coulter A, Bruster S (2002). The Picker Patient Experience Questionnaire: development and validation using data from in-patient surveys in five countries. Int J Qual Health Care.

[B22] Sitzia J (1999). How valid and reliable are patient satisfaction data? An analysis of 195 studies. Int J Qual Health Care.

[B23] Tanzania Ministry of Health (2005). Guideline for Reforming Hospitals at Regional and District Levels.

[B24] Franco LM, Bennett S, Kanfer R (2002). Health sector reform and public sector health worker motivation: a conceptual framework. Soc Sci Med.

[B25] Ross-Degnan D, Laing R, Santoso B, Ofori-Adjei D, Lamoureux C, Hogerzeil H (1997). Improving pharmaceutical use in primary care in developing counties: a critical review of experience and lack of experience. Presented at the International Conference on Improving Use of Medicines.

[B26] Howie SR, Hill SE, Peel D, Sanneh M, Njie M, Hill PC, Mulholland K, Adegbola RA (2008). Beyond good intentions: lessons on equipment donation from an African hospital. Bull World Health Organ.

[B27] Le Grand J (2006). Motivation, Agency, and Public Policy Of Knights and Knaves, Pawns and Queens.

[B28] Andersen HM (2004). "Villagers": differential treatment in a Ghanaian hospital. Soc Sci Med.

[B29] Chandler CIR, Chonya S, Mtei F, Reyburn H, Whitty CJM (2009). Motivation, money and respect: a mixed-method study of Tanzanian non-physician clinicians. Soc Sci Med.

[B30] Manzi F, Kida T, Mbuyita S, Palmer N, Gilson L (2004). Exploring the Influence of Workpace Trust over Health Worker Performance. Preliminary National Overview Report: Tanzania. Prepared for the Health Economics and Financing Programme, London School of Hygiene and Tropical Medicine.

[B31] Kanfer R (1999). Measuring Health Worker Motivation in Developing Countries. Major Applied Research 5, Working Paper 1.

[B32] Herzberg F, Mausner B, Snyderman B (1959). The Motivation to Work.

[B33] Dieleman M, Toonen J, Toure H, Martineau T (2006). The match between motivation and performance management of health sector workers in Mali. Hum Resour Health.

[B34] Cornwall A, Jewkes R (1995). What is participatory research?. Soc Sci Med.

[B35] Fitzpatrick R, Hopkins A (1983). Problems in the conceptual framework of patient satisfaction research: an empirical exploration. Sociol Health Illn.

[B36] Jenkinson C, Coulter A, Bruster S, Richards N, Chandola T (2002). Patients' experiences and satisfaction with health care: results of a questionnaire study of specific aspects of care. Qual Saf Health Care.

